# A Novel Hybrid Approach to Manage Mycotic Pseudoaneurysm Post-Renal Transplantation: Successful Graft Preservation

**DOI:** 10.3390/medicina61030521

**Published:** 2025-03-17

**Authors:** Ho Li, Yi-Chang Lin, Chien-Chang Kao, Pei-Jhang Chiang, Meng-Han Chou, Hui-Kung Ting, Yu-Cing Jhuo, Ming-Hsin Yang, Chih-Wei Tsao, En Meng, Guang-Huan Sun, Dah-Shyong Yu, Sun-Yran Chang, Chin-Li Chen, Sheng-Tang Wu

**Affiliations:** 1 Division of Urology, Department of Surgery, Tri-Service General Hospital, National Defense Medical Center, Taipei 114, Taiwan; liho0827@gmail.com (H.L.); peijhang@gmail.com (P.-J.C.); princecharmingben@gmail.com (M.-H.C.); wavinglibra1012@gmail.com (Y.-C.J.); yangming@ndmctsgh.edu.tw (M.-H.Y.);; 2 Division of Urology, Department of Surgery, Gangshan Branch of Zuoying Armed Forces General Hospital, Kaohsiung 820, Taiwan; 3 Division of Cardiovascular Surgery, Department of Surgery, Tri-Service General Hospital, National Defense Medical Center, Taipei 114, Taiwan

**Keywords:** renal transplantation, post-transplant pseudoaneurysm, endovascular intervention, femoral-femoral bypass, graft preservation, percutaneous transluminal angioplasty, coil embolization, kidney transplant complications, hybrid surgical approach

## Abstract

*Background and Objectives*: Post-transplant anastomotic pseudoaneurysms are a rare but serious complication of renal transplantation, typically requiring graft sacrifice. This case report demonstrates a novel hybrid approach for managing a mycotic pseudoaneurysm while preserving graft function. *Case report:* A 56-year-old male developed a pseudoaneurysm at the anastomotic site after cadaveric kidney transplantation, presenting with recurrent infections and declining renal function. Imaging confirmed the pseudoaneurysm. A hybrid strategy combining femoral–femoral bypass with a polytetrafluoroethylene graft, percutaneous transluminal angioplasty with stenting, and coil embolization was performed. *Results*: The intervention successfully isolated the pseudoaneurysm and preserved graft function. Post-procedure, serum creatinine levels improved, stabilizing at 2.3 mg/dL during follow-up. Imaging confirmed no residual flow in the aneurysm, and vascular complications were absent. *Conclusions*: This report highlights a successful combined surgical and endovascular approach for treating mycotic pseudoaneurysms, preserving graft function and restoring limb blood flow. This strategy offers a promising alternative for managing complex post-transplant complications, though long-term outcomes require further evaluation.

## 1. Introduction

Post-transplant pseudoaneurysms are a rare complication following renal transplantation, with an incidence of less than 1% [1]. Typically located at the anastomotic site, these pseudoaneurysms may arise from both infectious and non-infectious origins [2,3]. Although uncommon, they represent a significant complication, with risks to both graft and patient survival. Treatment modalities include open surgical repair, endovascular intervention, and ultrasound-guided percutaneous thrombin injection. Historically, treatment often necessitated allograft sacrifice despite the availability of various repair techniques [4]. In this report, we present a case of post-transplant anastomotic pseudoaneurysm in which allograft function was preserved using a combined approach of femoral–femoral bypass, percutaneous transluminal angioplasty (PTA) with stenting, and coil embolization.

## 2. Case Report

A 56-year-old male patient with end-stage renal disease began hemodialysis in 2020 and subsequently underwent cadaveric kidney transplantation (right kidney to left iliac fossa) on 5 December 2023. Postoperatively, he experienced multiple hospitalizations for urinary tract infections and episodes of bacteremia. Urine cultures identified Enterococcus faecium, Proteus mirabilis, and coagulase-negative Staphylococcus, while blood cultures consistently grew Pseudomonas aeruginosa. He received antibiotic therapy with agents including ceftazidime, piperacillin/tazobactam, and Fosfomycin at various stages.

**Figure 1:**
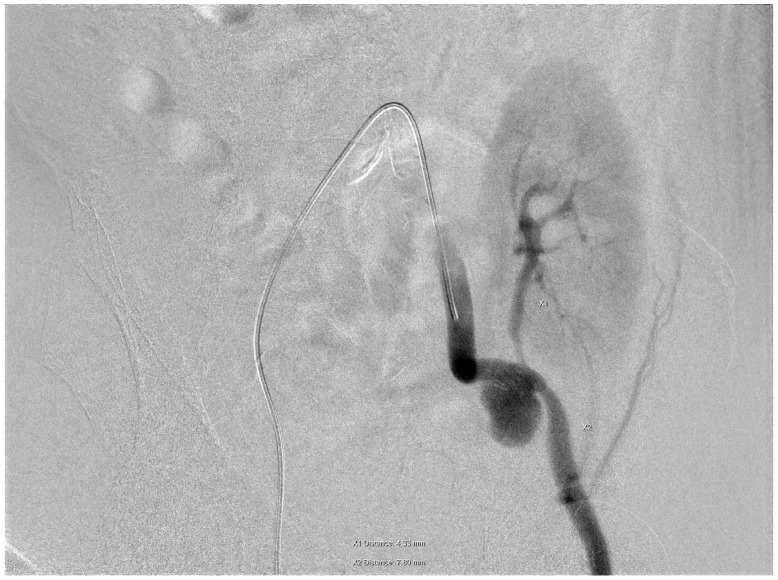
A pseudoaneurysm located at the anastomotic site on the left external iliac artery (EIA). Digital subtraction angiography (DSA) through the right common femoral artery demonstrated an EIA diameter of 7.8 mm distal to the anastomosis and a transplant renal artery (TRA) diameter of 4.3 mm.

The patient attended regular follow-up assessments post-transplant, with serum creatinine levels fluctuating between 2.3 and 3.4 mg/dL. In April 2024, his serum creatinine level increased to 4.8 mg/dL. A duplex ultrasound of the transplanted kidney identified a mass lesion, later confirmed via angiography to be a pseudoaneurysm located at the anastomotic site on the left external iliac artery (EIA). Digital subtraction angiography (DSA) through the right common femoral artery demonstrated an EIA diameter of 7.8 mm distal to the anastomosis and a transplant renal artery (TRA) diameter of 4.3 mm (Figure 1).

**Figure 2:**
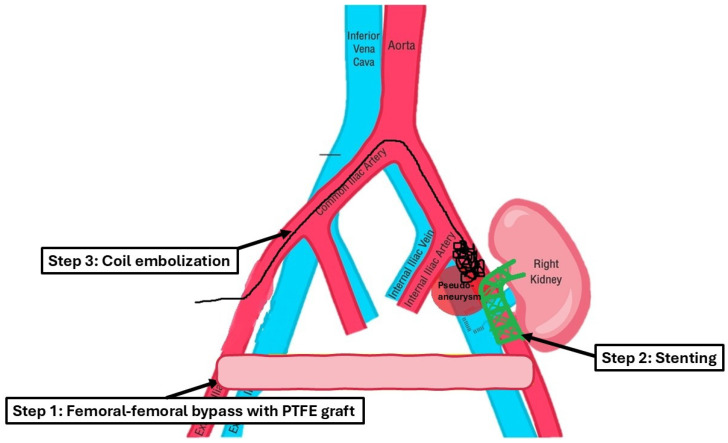
This schematic diagram demonstrates the approach used for a multi-step procedure.

**Figure 3:**
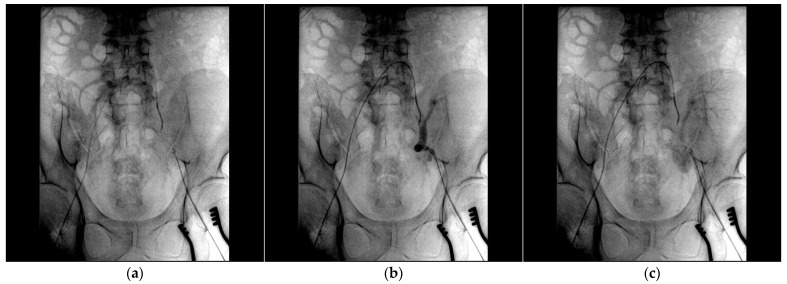
(**a**) The angiography catheter is placed in the left common iliac artery; (**b**,**c**) a large pseudoaneurysm projecting over the common and external iliac arteries is depicted.

**Figure 4:**
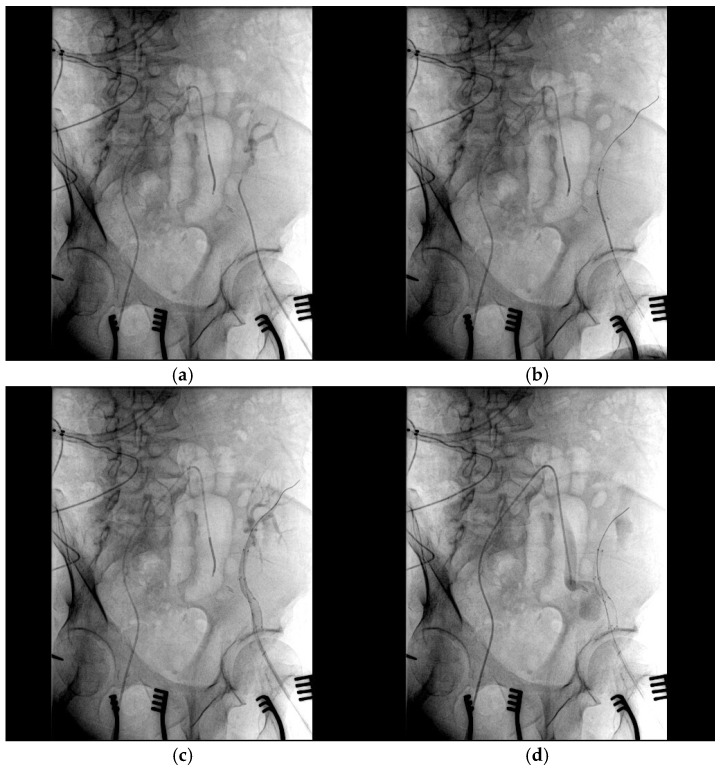
The stenting of both the renal artery of the graft kidney and the left external iliac artery: (**a**) The angiography catheter is placed in the renal artery of the graft kidney; (**b**) two covered 6.0 × 50 mm and 8.0 × 50 mm stents (Viabahn, WL Gore & Associates, Flagstaff, AZ, USA) were placed in the left external iliac artery and renal artery of the graft kidney. (**c**) Stents excluding the inflow to the pseudoaneurysm; (**d**) the blood flow from the common iliac artery was unable to pass into the graft kidney after stenting.

**Figure 5:**
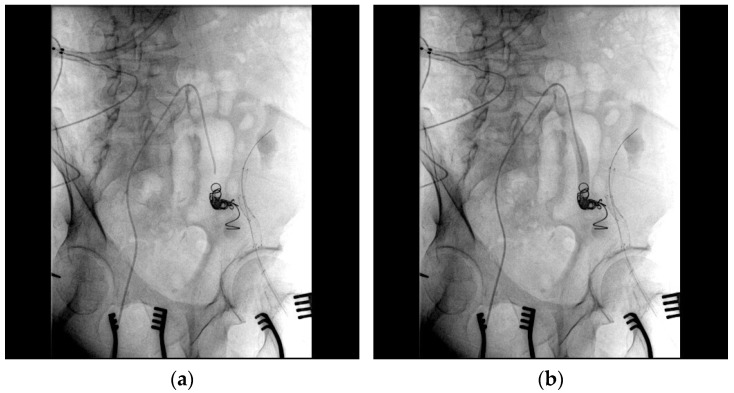
(**a**) Coil embolization of the left pseudoaneurysm and left common iliac artery was performed; (**b**) angiography verified no blood flow through the pseudoaneurysm.

After assessing the feasibility of an interventional endovascular approach, a multi-step procedure was performed (Figure 2). Initially, a femoral–femoral bypass was established using an 8 mm Geotex polytetrafluoroethylene (PTFE) graft, ensuring robust blood flow through the vascular graft after end-to-side anastomosis. Next, the left common femoral artery was accessed percutaneously (Figure 3), followed by stenting of both the renal artery of the graft kidney and the left external iliac artery to exclude inflow to the pseudoaneurysm (Figure 4). Coil embolization of the left pseudoaneurysm and left common iliac artery was subsequently performed (Figure 5a). Angiography verified no blood flow through the pseudoaneurysm (Figure 5b).

**Figure 6:**
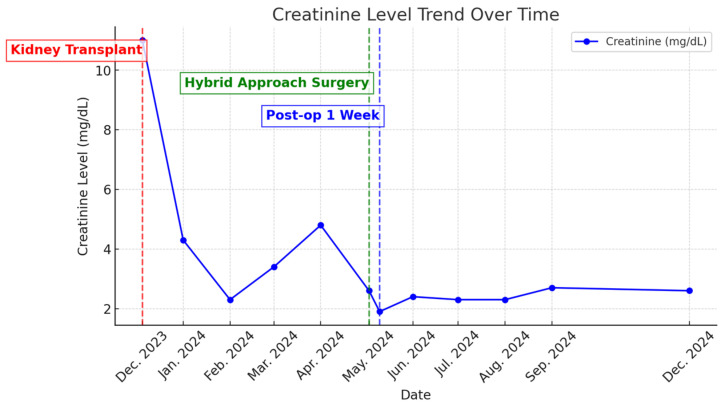
Creatinine level trend over time.

One week post-intervention, the patient’s serum creatinine had decreased to 1.9 mg/dL. At the 3-month and 6-month follow-ups, serum creatinine levels were 2.3 mg/dL and 2.6 mg/dL, respectively (Figure 6). Duplex ultrasound at 3 months showed a maximum arterial blood flow velocity of 83 cm/s in the TRA and 13 cm/s in the renal interlobular artery, with no detectable flow in the aneurysm. No vascular complications were observed at the 3-month and 6-month follow-ups.

## 3. Discussion

An arterial anastomotic pseudoaneurysm is an uncommon complication in renal transplant recipients, affecting fewer than 1% of cases. Presentations can include compromised renal graft function, ischemic or thrombotic symptoms in the ipsilateral limb, abdominal pain, prolonged fever, and unexplained anemia, which may indicate an extrarenal pseudoaneurysm [5]. The indications for intervention in pseudoaneurysms and the selection of an optimal treatment technique remain points of debate. While some of the literature supports conservative management for small, asymptomatic pseudoaneurysms [6], intervention is generally advised for those that are symptomatic, larger than 2.5 cm, rapidly enlarging, or when infection is implicated [2,6].

Traditionally, pseudoaneurysms were managed through open surgical repair, although this approach often led to allograft loss [1,4,7]. Recently, endovascular options such as coil embolization, stenting, and thrombotic agents have emerged as alternative treatments. Although promising for some, previous studies have often regarded endovascular repair as a preliminary intervention prior to transplant nephrectomy, with the transplanted kidney ultimately unable to be preserved [1,4,8]. As documented by Bracale et al. [6], among five cases of mycotic pseudoaneurysm, one patient underwent an attempted endovascular repair with coil embolization and stent placement. However, in an effort to maintain patency of the transplanted renal artery, the pseudoaneurysm may not have been adequately excluded. As a result, recurrence was observed within a short period, and, ultimately, none of the five patients were able to retain their transplanted kidney.

Only in recent reports has endovascular repair successfully preserved graft function [4]. Among the three cases reported by Anders et al. [4], one patient successfully preserved graft function following endovascular repair. This success can be attributed to the location of the pseudoaneurysm, which was confined to the renal artery rather than the anastomotic site. Therefore, a purely endovascular approach alone may not have effectively excluded the anastomotic pseudoaneurysm while preserving adequate blood flow to the transplanted kidney.

We conducted a comprehensive search of the PubMed/MEDLINE and Google Scholar databases (1975–2024) to identify all relevant studies on mycotic pseudoaneurysms following renal transplantation. We reviewed and summarized the different treatment approaches and their associated outcomes (Table 1).

When considering the treatment approach for this case, our primary objective was to preserve the kidney graft, given the scarcity of deceased donor renal transplants in our region [47]. Treatment options included open surgical repair; however, performing open surgery after renal transplantation is particularly challenging due to altered anatomy and extensive scarring from the initial transplant procedure [48]. Given these complexities, less invasive endovascular techniques have emerged as a promising alternative for managing infected pseudoaneurysms following renal transplantation [4,6,8].

However, the efficacy of these newer approaches in the specific setting of infected post-transplant pseudoaneurysms remains to be fully validated [6]. Overall, the management of these complex cases necessitates a multidisciplinary approach at specialized centers to optimize outcomes and maximize graft preservation whenever feasible [49].

In this particular case, however, the location of the pseudoaneurysm precluded the sole use of stenting and coil embolization, as these approaches would have risked compromising perfusion to the lower limb and the transplanted kidney. Given these considerations, the rationale for a hybrid approach was to achieve a balance between infection control, vascular integrity, and graft preservation as follows:

- Bypass grafting ensured continuous perfusion to the lower limb,
mitigating the risk of ischemic complications;
- Endovascular stenting minimized surgical trauma while effectively
excluding the pseudoaneurysm;
- Coil embolization provided an additional layer of pseudoaneurysm
isolation, further enhancing treatment efficacy.

Similar to the hybrid approach described by Buimer et al. [50], our strategy integrated both open and endovascular techniques. However, a key distinction in our case was the presence of a mycotic pseudoaneurysm, which introduced additional complexity due to the risk of persistent infection and reinfection. To enhance pseudoaneurysm isolation and effectively eliminate the infectious source, we combined endovascular stenting with coil embolization.

Furthermore, unlike Buimer et al., who performed a bypass from the common iliac artery to the renal transplant artery, our approach utilized a bilateral femoral artery bypass. This modification eliminated the need for temporary occlusion of the renal transplant artery during surgery, thereby preventing ischemic insult to an already compromised transplanted kidney. By maintaining continuous perfusion throughout the procedure, our approach sought to optimize graft preservation and improve overall patient outcomes.

Notably, endovascular repair is assumed to be not suitable in cases of mycotic pseudoaneurysm due to the high risk of reinfection and complications [44,51]. Although data on mycotic pseudoaneurysms following renal transplantation remain limited, endovascular treatment has emerged as a viable and effective approach for managing infected pseudoaneurysms across various anatomical locations. Multiple studies have reported successful outcomes with endovascular interventions, particularly when combined with targeted antibiotic therapy [8,52,53,54,55]. The efficacy of endovascular treatment appears to be enhanced when integrated with appropriate medical management, such as long-term antibiotic therapy or immunosuppressive regimen optimization [8,52,55].

Overall, endovascular treatment offers several advantages over open surgical repair for infected pseudoaneurysms, including reduced morbidity and mortality rates [55,56,57]. However, close follow-up remains crucial to detect potential complications such as persistent infection or recurrence [52,55].

In this case, endovascular stenting and coiling were implemented to isolate a mycotic pseudoaneurysm, and a femoral–femoral bypass was established to ensure adequate blood supply to the left lower extremity. The short-term clinical outcomes in this case are encouraging; however, complication and recurrence rates remain key concerns for long-term follow-up. Previous reports have indicated that coil embolization may fail to achieve permanent thrombosis of a false aneurysm in some cases, as described by Koo et al. in a renal transplant recipient [10].

Although data on the recurrence rate of mycotic pseudoaneurysms following endovascular treatment in renal transplant recipients are limited, studies on pseudoaneurysms from other etiologies, such as Behçet’s disease-related arterial pseudoaneurysms, have reported an 18.8% complication rate in the endovascular treatment group, including stent-graft occlusions and access site pseudoaneurysms, with a cumulative primary patency rate of 89% at 24 months [58,59].

In light of these findings, while endovascular treatment of infected pseudoaneurysms has demonstrated effectiveness, complication and recurrence rates remain variable and are influenced by factors such as underlying pathology, lesion location, and adjunctive therapies. Proper patient selection, meticulous perioperative management, and long-term follow-up are crucial for achieving optimal outcomes. Regular imaging surveillance and prolonged antibiotic therapy are essential to minimize reinfection risk and prevent vascular complications. Doppler ultrasound has been shown to be highly specific for diagnosing transplanted renal artery pseudoaneurysms, suggesting that early detection may improve clinical outcomes [60].

Overall, this patient initially experienced delayed graft function (DGF) following transplantation. With successful treatment with mycotic pseudoaneurysm and structured follow-up, we anticipate that the graft may achieve survival and functional recovery similar to typical cases of DGF [61].

## 4. Conclusions

In our view, optimal management of pseudoaneurysm should be customized to the patient’s condition. This case highlights the use of a specific hybrid approach that successfully preserved graft function in a mycotic pseudoaneurysm. While this approach appears promising, it remains single-case evidence. Further observation of long-term outcomes will be essential in subsequent follow-up studies.

## Figures and Tables

**Table 1:** A review of the literature on mycotic pseudoaneurysms following renal transplantation.

Intervention	Author	Year	Number/Treatment	Outcome
Open surgery (Including transplantectomy)	Kyriakides [9]	1976	8/Tx 1/SR	2/death 6/graft loss
	Koo [10]	1999	1/Tx	1/graft loss
	Battaglia [11]	2000	2/Tx	2/graft loss
	Garrido [12,13]	2003	2/Tx	1/death 1/graft loss
	Saidi [14]	2004	2/Tx	2/graft loss
	Laouad [10,13]	2005	4/Tx	1/death 3/graft loss
	Zavos [8]	2005	2/Tx	2/graft loss
	Eng [14,15]	2006	4/Tx	4/graft loss 2/death
	Nguan [15,16]	2006	1/Tx	1/graft loss
	Orlando [17,18]	2009	2/Tx	2/death
	Liu [19]	2009	1/Tx	1/graft loss
	Osman [20]	2009	1/EVS and Tx	1/graft loss
	Wang [21]	2009	4/Tx	4/graft loss
	Bracale [6]	2009	8/Tx 3/EVS and Tx 1/SR and replantation	8/graft loss 3/death after OP 1/graft preserved
	Bozkurt	2010	2/Tx	2/graft loss
	Berglund [22]	2011	1/SR	1/graft preserved
	Akhtar [23]	2011	1/Tx	1/graft loss
	Lee [24]	2011	1/Tx	1/graft loss
	Minz [25]	2011	2/Tx	1/death 1/graft loss
	Polat [26]	2011	1/Tx	1/graft loss
	Kountidou [27]	2012	1/SR	1/graft preserved
	Leonardou [28]	2012	4/EVS and Tx	4 graft loss
	Santangelo [29]	2013	1/SR and replantation 4/Tx 1/EVS and Tx	5/graft loss 1/graft preserved
	Chandak [30]	2014	1/SR	1/graft preserved
	Debska-Slizien [31]	2015	2/Tx	2/death after OP
	Madhav [32]	2015	1/SR	1/graft preserved
	Patrono [3]	2015	2/Tx 1/SR	2/graft loss 1/graft preserved
	Zhao [33]	2016	2/EVS and Tx	2/graft loss
	Fananapazir [34]	2016	3/Tx	3/graft loss
	Berger [35]	2017	1/Tx	1/graft loss
	Chung [36]	2017	1/Tx	1/graft loss
	Lin [37]	2017	1/Tx 1/SR	1/graft loss 1/graft preserved
	Ministro [38]	2017	2/SR	2/graft preserved
	Liu [39,40]	2018	5/SR	5/graft preserved
	Khalil [41]	2024	1/Tx	1/graft loss
	Horn [42]	2024	1/SR	1/graft preserved
Endovascular repair	Koo [10]	1999	1/EVC	1/graft preserved
	Peel [5]	2003	1/EVC	1/graft preserved
	Poels [43]	2007	1/EVS + thrombin	1/graft preserved
	Berger [35]	2017	1/EVS	1/graft preserved
	Che [44]	2014	1/EVS	1/graft preserved
	Patil [45]	2015	1/EVS	1/graft preserved
	Fananapazir [34]	2016	1/EVC	1/graft preserved
	Hassanein [46]	2024	1/EVS	1/graft preserved

Tx: transplantectomy; SR: surgical repair; EVS: endovascular stenting; EVC: endovascular coiling.

## Data Availability

The clinical data (patient data, pictures, etc.) were all retrieved with the consent of the patients and the hospital involved. All referential data were retrieved with consent. Further inquiries can be directed to the corresponding authors.
